# Permeation characteristics of tetracyclines in parallel artificial membrane permeation assay II: Effect of divalent metal ions and mucin

**DOI:** 10.5599/admet.797

**Published:** 2020-05-01

**Authors:** Sachika Yamauchi, Daisuke Inoue, Kiyohiko Sugano

**Affiliations:** Molecular Pharmaceutics Lab., College of Pharmaceutical Sciences, Ritsumeikan University, 1-1-1, Noji-higashi, Kusatsu, Shiga 525-8577, Japan

**Keywords:** artificial membrane, permeability, phospholipid, tetracycline, metal, cation, mucin

## Abstract

The bioavailability of tetracyclines is markedly decreased when co-administered with antacids, milk, or food containing Ca^2+^. Previously, it was suggested that the effective intestinal permeation of tetracycline (TC) was decreased due to Ca^2+^ linked mucin binding in the mucosal side. In the present study, we investigated the effect of Ca^2+^, Mg^2+^, and mucin on the membrane permeation of six tetracyclines (TC, oxytetracycline (OTC), minocycline (MINO), doxycycline (DOXY), demeclocycline (DMCTC), and chlortetracycline (CTC)). The membrane permeability values (P_e_) of tetracyclines were measured by the parallel artificial membrane permeation assay (PAMPA) using soybean lecithin – decane (SL–PAMPA) and octanol (OCT–PAMPA) membranes. In SL–PAMPA, Ca^2+^ markedly decreased the P_e_ values of all tetracyclines. In OCT–PAMPA, Ca^2+^ increased the P_e_ values of TC, CTC, and DMCTC, but not DOXY, OTC, and MINO. Mg^2+^ decreased the P_e_ values of all tetracyclines in both SL–PAMPA and OCT–PAMPA (except for CTC in OCT–PAMPA). The addition of mucin had little or no effect in all cases. In contrast to the previously suggested mechanism, the results of the present study suggested that Ca^2+^ chelate formation decreased the membrane permeation of tetracyclines, irrespective of Ca^2+^ linked mucin binding. Molecular speciation analysis suggested that the permeation of TC – metal chelates was negligibly small in SL-PAMPA.

## Introduction

Co-administration of multivalent metal ions reduces the bioavailability of various drugs, such as tetracyclines, fluoroquinolones, HIV-integrase inhibitors, and platelet-stimulating agents [1–4]. For example, the bioavailability of tetracyclines is markedly decreased when co-administered with antacids, milk, and food containing Ca^2+^ [4–10]. It is generally accepted that chelate formation between tetracycline (TC) and Ca^2+^ is behind the observed decrease in the bioavailability of tetracyclines [11–16]. Chelate formation of tetracyclines has been extensively investigated (Ref. [17] and references therein). However, the exact mechanism of the Ca^2+^ effect on the bioavailability of tetracyclines has not been clear.

Several ex-vivo and in-situ studies have shown that divalent metal ions, such as Ca^2+^, Mg^2+^, and Fe^2+^, reduce the intestinal wall permeation of tetracyclines [18–22]. In 1968, Kakemi *et al*. investigated the effect of Ca^2+^ on the effective intestinal wall permeation of tetracycline (TC) using the rat small intestine [21, 22]. They also measured the isopentanol – buffer partition coefficient as a surrogate of passive transcellular membrane permeability without the interference from mucin. They found that Ca^2+^ decreased the effective intestinal wall permeation of TC in the rat ex-vivo experiment, but increased the partition coefficient of TC. They also found that TC bound to the intestinal mucin layer in the presence of Ca^2+^. Based on these observations, they suggested that Ca^2+^ linked mucin binding decreased the TC concentration available for membrane permeation, resulting in a decrease in the effective intestinal wall permeation. Schumacher and Linn also reported that Ca^2+^ increased the transfer rate of TC from the aqueous phase to the octanol phase [23]. However, it is questionable whether these alcohol systems could be a good surrogate model for investigating the effect of divalent metal ions on the membrane permeation of drugs. Since divalent metal ions may affect cellular integrity, mucin-free cell-based systems such as Caco-2 have rarely been used to examine the effects of multivalent metal ions on membrane permeation [24, 25].

The parallel artificial membrane permeation assay (PAMPA) has been widely used to assess the passive membrane permeation of a drug [26–29]. Phospholipid-based artificial membranes are most commonly used with PAMPA. PAMPA permeability correlates with the in vivo and cellular permeation of drugs better than the octanol-buffer partition coefficient [27, 28]. Recently, we reported the permeation characteristics of tetracyclines in a phospholipid-based PAMPA [30]. Only a weak correlation was observed between the PAMPA permeability (*P*_e_) and the octanol-buffer partition coefficients (log *D*_oct_) for tetracyclines, suggesting that chemoselectivity differs between these systems. However, the effects of divalent metal ions on the PAMPA permeation of tetracyclines have been unknown.

**Figure 1.**
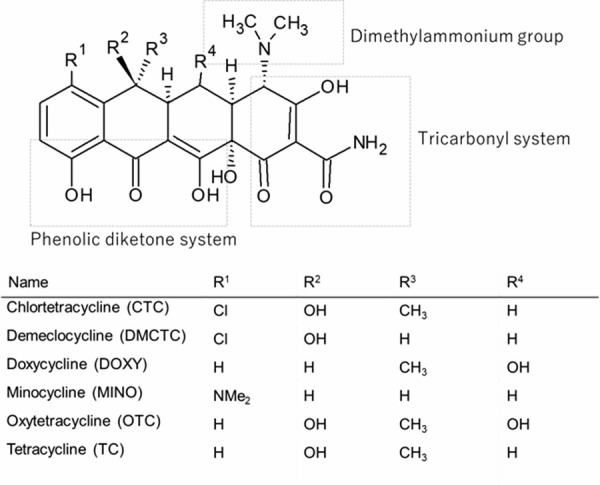
Chemical structures of tetracyclines

The purpose of the present study was to investigate the effect of Ca^2+^, Mg^2+^, and mucin on the phospholipid-based PAMPA permeation of tetracyclines. Six tetracycline derivatives were used in this study (Figure 1). The physicochemical properties of these tetracyclines have been summarized in Table 1 [31–33].

## Experimental

### Materials

Tetracycline hydrochloride (TC), decane, calcium dichloride, magnesium dichloride, octanol, pig stomach mucin, and 8 M NaOH were purchased from Wako Pure Chemical Industries, Ltd (Osaka, Japan). Oxytetracycline hydrochloride (OTC), minocycline hydrochloride (MINO), and doxycycline hyclate (DOXY) were purchased from TCI (Tokyo, Japan). 2-Morpholinoethanesulfonic acid (MES) was purchased from Dojindo laboratories (Tokyo, Japan). Demeclocycline hydrochloride (DMCTC) and chlortetracycline hydrochloride (CTC) were purchased from LKT Labs, Inc (MN, USA). Soybean lecithin (SLP-white) was provided by Tsuji Oil Mills co., Ltd (Mie, Japan).

### PAMPA assay

The PAMPA sandwich was consisted of a 96 well filter plate (hydrophobic PVDF, 0.45 μm) and a PAMPA acceptor plate (Merck Millipore, MA, USA). Before forming the PAMPA sandwich, the bottom (acceptor) plate was filled with 300 μL of a 50 mM MES buffer (pH 6.5). The filter of the top (donor) compartment was coated with 5 μL of a 10% soybean lecithin (SL) – decane solution or octanol. A drug solution (0.5 mM, 200 μL) with or without a divalent metal ion (5 mM) and/or mucin (1%) in the same buffer was added to the donor compartment. The PAMPA sandwich was then incubated for 3 h at 37 °C. After incubation, 150 μL of both the donor and acceptor solutions were transferred to a UV plate. The concentrations of tetracyclines were measured at 360 nm. The PAMPA permeability (*P*_e_) was calculated by the following equation [34].


(1)

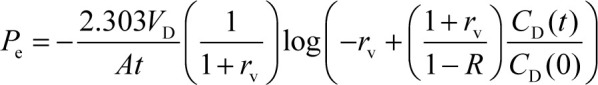




(2)

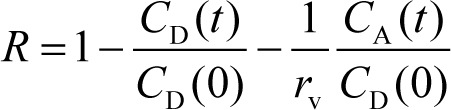




(3)

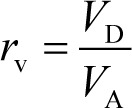



where *P*_e_ is the effective permeation coefficient (cm/s), *A* is the filter surface area (0.266 cm^2^), *V*_D_ and *V*_A_ are the volumes (mL) in the donor and acceptor phase, *t* is the incubation time, *C*_D_(*t*) is the concentration of a drug in the donor phase at time *t*, *R* is the membrane retention factor, and *r*_v_ is the volume ratio. We confirmed that the phospholipid - decane membrane is stable during the experimental period (no leakage of impermeable substrate) (data not shown).

## Results and discussion

Previously, we reported that the *P*_e_ value of TC was markedly affected by the composition of phospholipids in PAMPA [30]. In this study, a soybean lecithin (SL, 10%) – decane membrane (SL–PAMPA) was used because it most likely mimics the intestinal membrane [34]. The soybean lecithin contained phosphatidylcholine (24-32%), phosphatidylethanolamine (20-28%), phosphatidylinositol (12-20%), phosphatidic acid (8-15%), and lysophosphatidylcholines (1–5%) (based on the product information provided by the manufacturer). In addition, an octanol membrane (OCT–PAMPA) [35, 36] was also used because Ca^2+^ was reported to increase the log *D*_oct_ of TC [23]. Since Ca^2+^ interacts with phosphate and citrate ions, MES buffer was used in this study. The concentration of Ca^2+^ was set to 5 mM based on the standard of the daily intake in food (600 mg) [37] and the gastrointestinal fluid volume in the fed state [38]. We previously reported that the *P*_e_ value of TC in SL-PAMPA was not affected by the ionic strength up to 2 mol/L (adjusted by NaCl) [34].

**Figure 2.**
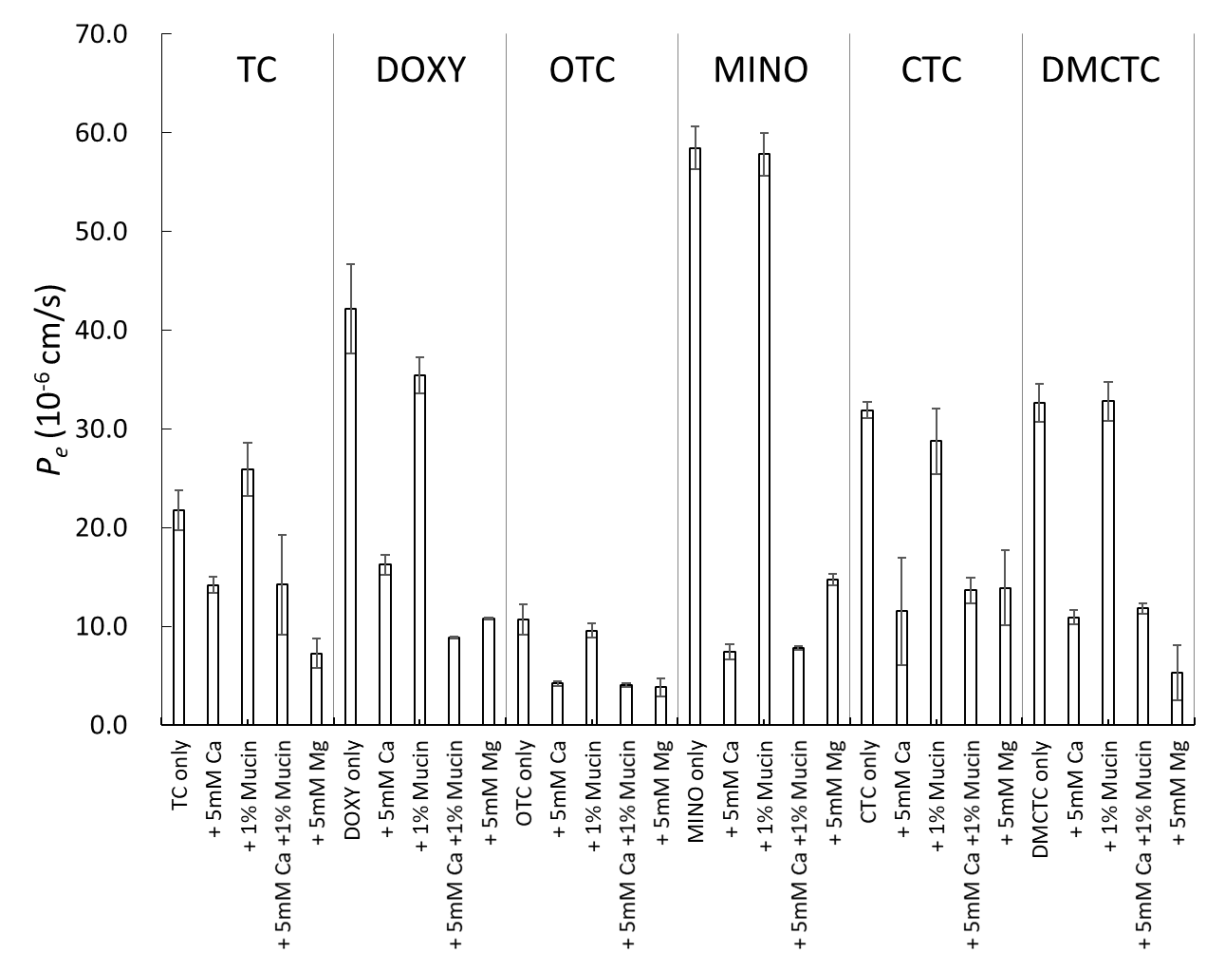
Effect of additives on SL–PAMPA permeation of tetracyclines (mean ± SD, n = 3 - 6).

In SL–PAMPA, Ca^2+^ and Mg^2+^ markedly decreased the *P*_e_ values of all tetracyclines investigated in this study, whereas mucin showed little or no effect (Figure 2). These results suggest that, in contrast to the previous suggestion based on the alcohol–water partition coefficient [21–23], Ca^2+^ chelate formation decrease the membrane permeation of tetracyclines, irrespective of Ca^2+^ linked mucin binding.

**Figure 3.**
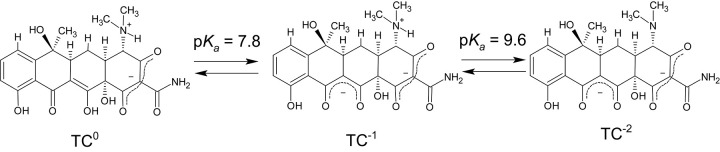
Ionization states of tetracycline (TC) at the neutral pH region. The macro p*K_a_* value and major molecular species are shown in this figure.

Molecular speciation analysis was performed to elucidate the effect of Ca^2+^ and Mg^2+^ on the SL- PAMPA permeation of tetracyclines. The details of molecular speciation analysis have been reported by Werner et al [39]. Tetracyclines and divalent metal ions can form a chelate with various stoichiometries (2:1, 1:1, 1:2), depending on the ionization state of tetracyclines and metal ion species [17, 40–44]. In this analysis, macro p*K*_a_ and major molecular species are considered [45, 46]. The fraction of each molecular species (L^0^, L^-1^, L^-2^, M^2+^L^-1^, M^2+^L^-2^: L = tetracyclines, M = metal) (Figure 3) was calculated from the p*K*_a_ values and the metal ion association constants (*K*_ML_ = [L^z^M^2+^]/([M^2+^][L^z^], z = -1, -2) of tetracyclines (Tables 1 and 2) [17, 39, 47]. In the neutral pH region, tetracyclines mainly exist as an equilibrium between a charge–neutral form (L^0^), and negatively charged forms (L^-1^_,_ L^-2^) (Figure 3) [31, 40, 47]. Even though L^0^ does not bind to the metal ions [47], Ca^2+^ and Mg^2+^ reduce the fraction of L^0^ (*f*_L0_) at pH 6.5 by shifting the equilibrium (Table 3). The reduction of *f*_L0_ corresponded to that of *P*_e_, except for the Ca^2+^ effect on OXY permeability, suggesting that the SL-PAMPA membrane is impermeable to M^2+^L^-1^. The pH - *P*_e_ relationship in our previous study [30] suggested that TC^0^, but not TC^-1^, predominantly permeates the SL-PAMPA membrane. However, further investigation is needed to better understand the effect of metal ions on tetracycline membrane permeation. The *K*_ML_ values reported in the literature show large variation [17, 39, 47]. The *f_L0_* value is especially sensitive to the *K*_ML_ value of M^2+^L^-1^. In addition, M^2+^L^-1^ chelates may have different stoichiometry (1: 1 or 1: 2) [17, 39, 47]. Micro speciation with micro p*K*_a_ values is required to decouple the contributions of uncharged and zwitterionic forms in L^0^ [45].

**Figure 4.**
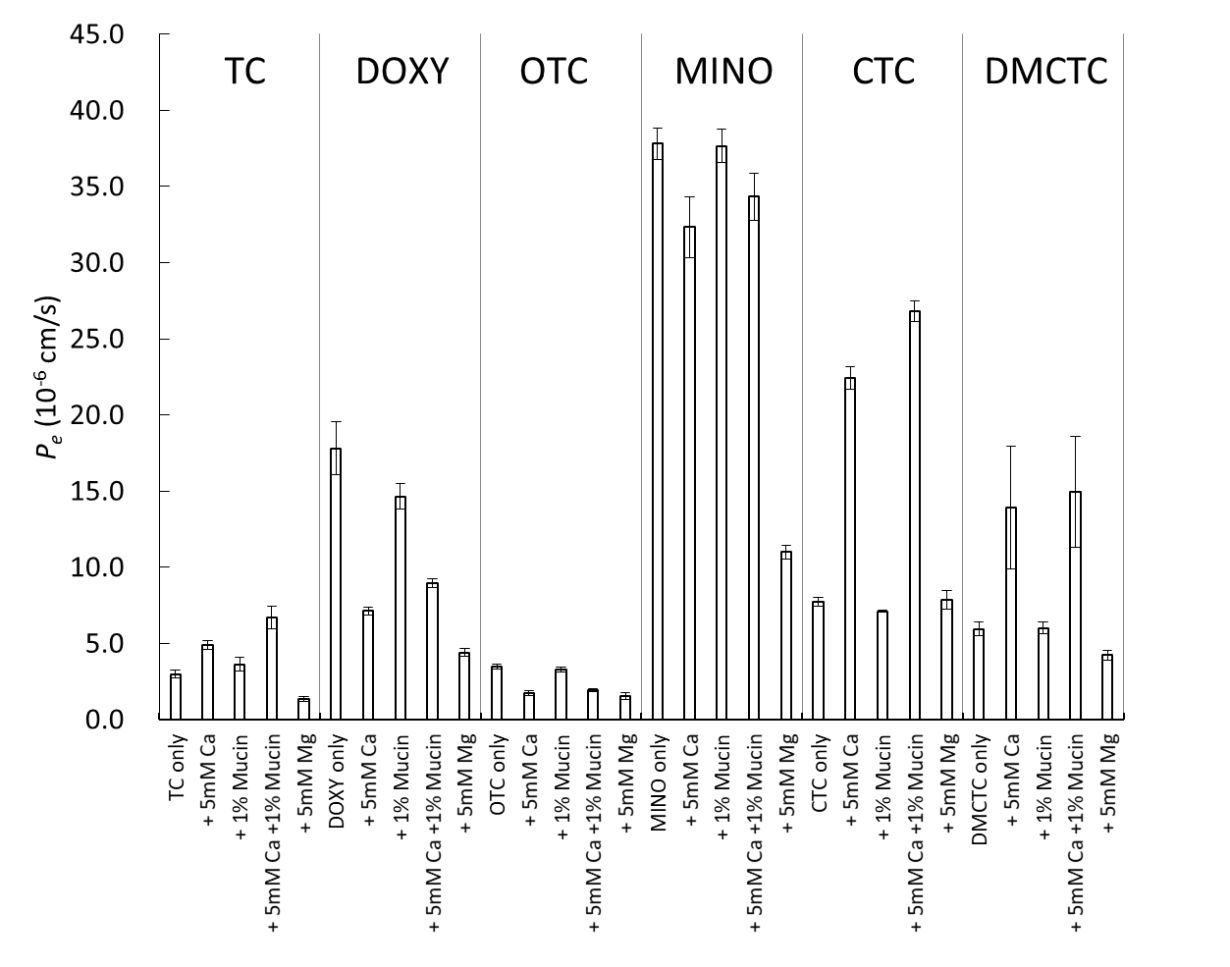
Effect of additives on OCT–PAMPA permeation of tetracyclines (mean ± SD, n = 3 - 6).

**Figure 5.**
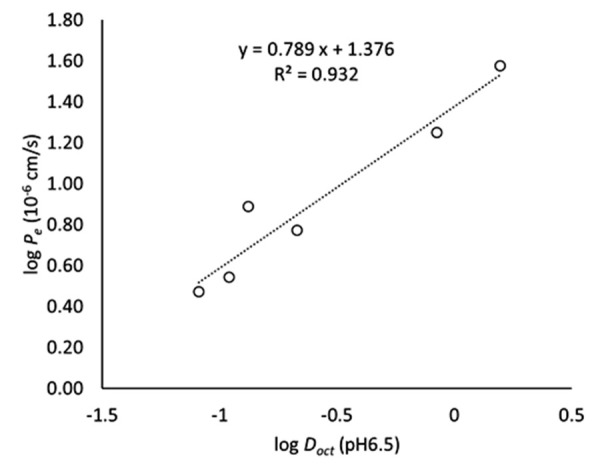
Correlation between log *D*_oct_ and log *P*_e_ of tetracyclines in OCT–PAMPA. The log *D*_oct_ values were taken from the literatrue [30].

In OCT–PAMPA, Ca^2+^ increased the *P_e_* values of TC, CTC, and DMCTC (Figure 4). This result is in good agreement with the previous studies investigating the Ca^2+^ effect on the alcohol – water partition coefficient of TC (octanol and isopentanol) [21–23]. Interestingly, Ca^2+^ affected SL–PAMPA and OCT–PAMPA in the opposite direction for TC, CTC, and DMCTC, but in the same direction for DOXY, OTC, and MINO. On the other hand, Mg^2+^ decreased the *P_e_* values of all tetracyclines in OCT–PAMPA except for CTC (no effect). These results suggest that it could be inappropriate to use octanol as a surrogate of a phospholipid membrane for investigating the effect of divalent metal ions. In OCT-PAMPA, the octanol phase could contain water molecules in reverse micelle structures [48]. This may facilitate the permeation of charged species, such as the TC – metal chelates. In similar to SL–PAMPA, the addition of mucin did not affect the *P_e_* values in OCT–PAMPA, suggesting that there is no interaction between tetracyclines and mucin. As expected, there is a good correlation between log *D_oct_* and log *P_e_* in OCT–PAMPA (Figure 5) [35, 36].

Clinically, co-administration of food and milk has been reported to decrease the bioavailability of tetracyclines (Table 4) [4–10]. The effect of food and milk on bioavailability is greater for TC and OXY, but relatively small for DOXY and MINO [4]. However, in the present study, the percent reduction of *P*_e_ by Ca^2+^ was smallest for TC (MINO (87%) > DMCTC (67%) ≈ CTC (64%) ≈ DOXY (62%) ≈ OXY (61%) > TC (35%)). The *P*_e_ values of MINO and DOXY are higher than that of the other tetracyclines. In addition, after oral administration, MINO and DOXY are almost completely absorbed, whereas TC, OXY, CTC and DMCTC are incompletely absorbed [4]. Therefore, the reduction of *P*_e_ by Ca^2+^ may have less impact on the bioavailability of MINO and DOXY. Barza et al. reported that, after the administration of tetracyclines with milk into the ileal loop in dogs, the remaining fraction in the luminal contents is DOXY >> OXY ≈ MINO ≈ TC [49]. Lipophilicity may play a role in food and milk binding. The balance of metal ion chelating, food/ milk binding, and membrane permeation may determine the extent of food and milk effects. Interestingly, the effects of metal ions and pH [30] on the bioavailability of TC to *E.coli* are similar to that on SL-PAMPA permeation [50].

We could not find any plausible chemical structural elucidation for the differences among tetracyclines regarding the effects of Ca^2+^ and Mg^2+^. Tetracyclines can easily modify their tautomerism in response to various chemical environments [44]. Metal binding to anionic phospholipids in the SL-PAMPA membrane may be another possible mechanism to reduce the permeation of tetracyclines. In our previous study, the addition of an anionic lipid neutralizer (tetrahexylammonium) did not affect the permeation of TC in SL-PAMPA, suggesting that the ionic interaction with anionic phospholipids do not facilitate the permeation of TC [30]. Further investigation is required to clarify the interactions among tetracyclines, metal ions, and phospholipids. We are currently investigating the effects of metal ions on the SL-PAMPA permeation of structurally diverse drugs.

## Conclusion

In contrast to the previously suggested mechanism [21, 22], in this study, Ca^2+^ chelate formation decreased the membrane permeation of tetracyclines, irrespective of Ca^2+^ linked mucin binding. Ca^2+^ affected the *P*_e_ values in SL–PAMPA and OCT–PAMPA in the opposite direction for some tetracyclines. SL–PAMPA can be a simple tool to qualitatively evaluate the effect of multivalent metal ions on the membrane permeation of drugs.

## Figures and Tables

**Table 1.** Physicochemical properties of tetracyclines

	*M*_W_	p*K*_a_	log *D*_oct_ (pH 6.5)^a^	p*K*_a_ Ref.
Chlortetracycline	479	3.3, 7.6, 9.3	-0.88	[31] ^b^
		3.25, 6.72, 8.84		[32] ^c^
Demeclocycline	465	3.4, 7.4, 9.4	-0.67	[31]^b^
Doxycycline	444	3.0, 8.0, 9.2	-0.08	[31] ^b^
		3.50, 7.25, 9.58		[32] ^c^
Minocycline	457	2.8, 5.0, 7.8, 9.5	0.20	[33] ^d^
Oxytetracycline	460	3.2, 7.5, 8.9	-0.96	[31] ^b^
		3.53, 7.25, 9.58		[32] ^c^
Tetracycline	444	3.3, 7.8, 9.6	-1.09	[31] ^b^
		3.35, 7.29, 9.88		[32] ^c^

^a^ Measured by a shake-flask method. Ref. [30].

^b^ Potentiometry (23 °C), ionic strength = 0.01 or 0.05 M.

^c^ Potentiometry (25 °C), ionic strength = 0.1 M.

^d^ Method not described in the literature.

**Table 2.** Association constants of Ca^2+^ and Mg^2+^ with tetracyclines (L = TC, CTC, or OXY)

Reactions	log *K*_ML_		
	TC	CTC	OXY
Ca^2+^ + L^-1^ ⇄ Ca^2+^L^-1^	3.4 ^a^, 3.0 ^b^	3.8 ^c^, 2.9 ^b^,	2.9 ^b^
Ca^2+^ + L^-2^ ⇄ Ca^2+^L^-2^	5.8 ^a^, 4.0 ^b^	5.9 ^c^, 3.9 ^b^,	3.8 ^b^, 4.9 ^c^
Mg^2+^ + L^-1^ ⇄ Mg^2+^L^-1^	3.9 ^a^, 3.5 ^b^	3.3 ^c^, 3.2 ^b^	3.3 ^b^
Mg^2+^ + L^-2^ ⇄ Mg^2+^L^-2^	4.1 ^a^, 4.2 ^b^	4.7 ^c^, 4.1 ^b^	4.3 ^b^, 5.2 ^c^

^a^ Ref. [39]

^b^ Ref. [47]

^c^ Ref. [17]

**Table 3.** Fraction of molecular species at pH 6.5 ^a^

Tetracyclines (L)	Metal ions (M)	Fraction of molecular species ^b^					Reduction %	
		L^0^	L^-1^	L^-2^	M^2+^- L^-1^	M^2+^- L^-2^	*f_L0_*	*P_e_*
TC	None	0.95	0.05	< 0.01	-^c^	-	-	-
	Ca^2+ d,e^	0.55	0.03	< 0.01	0.35	0.07	42	35
	Mg^2+ d,e^	0.33	0.02	< 0.01	0.65	< 0.01	65	67
CTC	None	0.93	0.07	< 0.01	-	-	-	-
	Ca^2+ d,e^	0.24	0.02	< 0.01	0.61	0.12	74	64
	Mg^2+ d,e^	0.53	0.04	< 0.01	0.42	0.02	43	56
OXY	None	0.91	0.09	< 0.01	-	-	-	-
	Ca^2+ d,e^	0.66	0.07	< 0.01	0.26	< 0.01	27	61
	Mg^2+ d,e^	0.47	0.05	< 0.01	0.47	0.02	49	64

^a^ Activity coefficients were assumed to be 1. See ref. [39] for details

^b^ L = TC, CTC, or OXY. M = Ca or Mg

^c^ Not applicable

^d^ 5.0×10^-3^ mol/L

^e^ The *K*_ML_ values were from Ref. [39], [17], [47] for TC, CTC, and OYX, respectively.

**Table 4.** Summary of food and milk effects on bioavailability of tetracyclines

Drugs	Percentage absorption, % ^a^	Bioavailability reduction, % ^b^		References for food and milk effect
		Food	Milk	
CTC	25–30	45 ^c^	NA ^d^	[9]
DMCTC	66	NA ^d^	70	[6]
DOXY	95	26 (3-49)	30 (9-53)	[10]
MINO	95-100	14 (2-51)	27 (8-61)	[6, 9, 10]
OXY	58	41 (4-77), 0 ^c, e^	83 (45-96)	[6, 9, 10]
TC	77–88	46 (13-73%), 72 ^c^	65	[5, 8–10]

^a^ Ref. [4]

^b^ In humans unless otherwise noted

^c^ In pigs

^d^ Data not available in the literature

^e^ Low bioavailability (3% in both fasted and fed pigs)
